# Parallel Reinforcement Pathways for Conditioned Food Aversions in the Honeybee

**DOI:** 10.1016/j.cub.2010.11.040

**Published:** 2010-12-21

**Authors:** Geraldine A. Wright, Julie A. Mustard, Nicola K. Simcock, Alexandra A.R. Ross-Taylor, Lewis D. McNicholas, Alexandra Popescu, Frédéric Marion-Poll

**Affiliations:** 1 Centre for Behaviour and Evolution, Institute of Neuroscience, Newcastle University, Newcastle upon Tyne NE1 7RU, UK; 2 School of Life Sciences, Arizona State University, Tempe, AZ 85287, USA; 3 School of Biology, Newcastle University, Newcastle upon Tyne NE1 7RU, UK; 4 Unité Mixte de Recherches Physiologie de l'Insecte: Signalisation et Communication 1272 Institut National de Recherches Agronomiques (INRA)-Université Pierre et Marie Curie, INRA-Versailles, route de Saint Cyr, 78026 Versailles Cedex, France; 5 AgroParisTech, Département Sciences de la Vie et Santé, 16 rue Claude Bernard, 75231 Paris Cedex, France

## Abstract

Avoiding toxins in food is as important as obtaining nutrition. Conditioned food aversions have been studied in animals as diverse as nematodes and humans [1, 2], but the neural signaling mechanisms underlying this form of learning have been difficult to pinpoint. Honeybees quickly learn to associate floral cues with food [3], a trait that makes them an excellent model organism for studying the neural mechanisms of learning and memory. Here we show that honeybees not only detect toxins but can also learn to associate odors with both the taste of toxins and the postingestive consequences of consuming them. We found that two distinct monoaminergic pathways mediate learned food aversions in the honeybee. As for other insect species conditioned with salt or electric shock reinforcers [4–7], learned avoidances of odors paired with bad-tasting toxins are mediated by dopamine. Our experiments are the first to identify a second, postingestive pathway for learned olfactory aversions that involves serotonin. This second pathway may represent an ancient mechanism for food aversion learning conserved across animal lineages.

## Highlights

► Learned food avoidances arise from two independent reinforcement pathways ► Detection of toxins requires the responses of multiple gustatory neurons ► Learned avoidance of odors associated with aversive taste involves dopamine ► Learned avoidance of odors associated with toxin ingestion involves serotonin

## Results and Discussion

### Honeybees Detect Toxins in Sucrose Solutions

**Figure 1:**
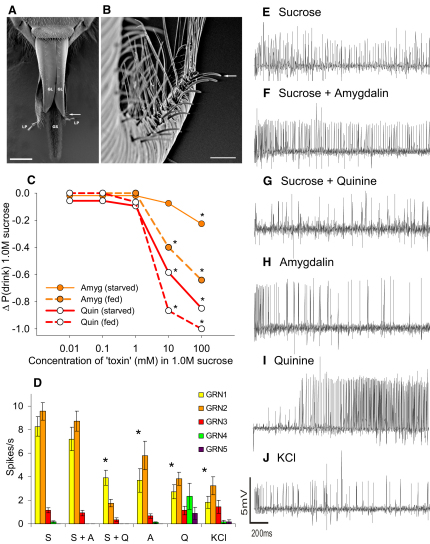
Honeybees Are More Sensitive to Quinine Than to Amygdalin in Sucrose Solution (A) The structure of the honeybee's proboscis revealed by scanning electron microscopy: the galea (GL) of the two maxillae, and the labium comprised of the two labial palps (LP) attached to the glossa (GS). The arrow indicates the end of the galea from which (B) was photographed. Scale bar represents 500 μm. (B) The dorsal view, looking down to the tip of the galea. The arrow indicates the first of the ten sensilla chaetica from which tip recordings were made. Scale bar represents 50 μm. (C) Honeybees were more likely to reject solutions containing quinine (logistic regression: χ_1_^2^ = 49.9, p < 0.001), and honeybees fed prior to testing were more sensitive to toxins in solution. n_quin_ = 30, n_amy_ = 30. Asterisks indicate where the response was significantly different to the 1.0 M sucrose control (least-squares multiple comparison tests, p < 0.05). The Δ value on the y axis is the deviation from the mean probability of drinking 1.0 M sucrose alone. (D) The rate of response of the neurons (GRN) in each sensillum depended on the stimulating solution. Each solution produced a distinct ratio of activity in the galeal GRN population. Asterisks indicate the stimuli with responses in GRN classes 1 and 2 that were significantly different to the sucrose control (t test, p < 0.05). n_S_ = 71, n_S+A_ = 43, n_S+Q_ = 35, n_A_ = 42, n_Q_ = 39, n_KCl_ = 23. Error bars represent ±standard error of the mean (SEM). (E–J) Two-second tip recordings were made from the galeal sensilla. Each voltage trace represents the following stimuli: 300 mM sucrose (E), 300 mM sucrose with 10 mM amygdalin (F), 300 mM sucrose with 10 mM quinine (G), 10 mM amygdalin (H), 10 mM quinine (I; see also Figure S1C), 1 mM KCl (J; the electrolyte used as the baseline conducting solution).

We examined whether honeybees could learn to associate an odor with the presence of a toxin in food, with the ultimate aim of identifying the neural mechanisms that underpin conditioned food aversions. We first established whether bees could preingestively detect toxins in food. Using a combination of behavioral and electrophysiological techniques, we tested the honeybee's sensitivity to two toxins, quinine and the almond-nectar toxin, amygdalin. To assess whether the gustatory sensilla on the proboscis (mouthparts) were sensitive to the presence of toxins (Figures 1A and 1B), we stimulated the antenna with 1.0 M sucrose to elicit the proboscis extension reflex (PER) [8] and then applied the sucrose-toxin solutions to the sensilla at the tip of the extended proboscis to measure whether the bee would drink. Because hunger state can influence an insect's sensitivity to toxins [9], we tested two groups: a group fed to satiety and a group starved for 24 hr prior to testing. The probability that a bee would refuse to drink the solution depended on the toxin and its dose (logistic regression: χ_4_^2^ = 36.1, p < 0.001) and on whether it had been fed to satiety prior to testing (Figure 1C; logistic regression: χ_4_^2^ = 22.1, p < 0.001). These data indicate that the honeybee's proboscis is more sensitive to quinine than to amygdalin. By force-feeding individual bees a 10 μl dose of sucrose-toxin solution for each concentration depicted in Figure 1C, we found that both quinine and amygdalin were toxic when consumed with an LC_50_ of 10 mM at 24 hr (see Figure S1 available online).

To investigate the mechanism of toxin detection, we made tip recordings from the ten most distally located gustatory sensilla on the galea of the proboscis (Figure 1B). The ratio of the responses of the gustatory receptor neuron (GRN) types housed in these sensilla could be used to identify the stimulating solution (Figure 1D; canonical discriminant analysis, first function; Table S1). Two GRN types (spike classes 1 and 2) responded vigorously to sucrose but also responded to stimulation with both toxins, albeit at a lower rate, especially when stimulated with solutions containing quinine (Figure 1D; Figure S1). In contrast, two different GRN types (spike classes 4 and 5) responded robustly when stimulated with quinine alone but rarely responded to amygdalin, sucrose, or stimulation with either of the sucrose-toxin solutions. In a subset of sensilla stimulated with quinine, the class 4 GRNs produced a distinctive “deterrent cell” response observed in other insects; this response started with a latency period followed by rapid bursting (Figure 1I; Figure S1C) [10]. Another subset exhibited large oscillations in voltage (12 out of 45) and short-duration bursts or abnormal spikes (previously called injury potentials; Figure S1C) [11]. Our results clearly show that toxin detection involves specific temporal patterns of activity in many types of GRN [10], including those that respond most to sucrose, implying that an insect's ability to sense toxins is not a labeled line encoded via the responses of specific toxin-detecting neurons.

### Pre- and Postingestive Processes Contribute to Avoidance Learning

**Figure 2:**
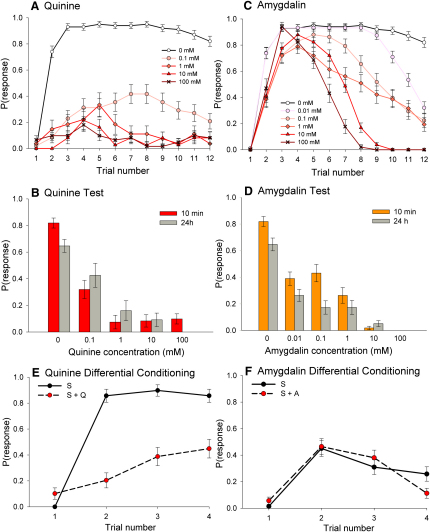
Honeybees Use Both Pre- and Postingestive Mechanisms to Learn to Avoid Toxins in Sucrose Solution (A) Low levels of PER indicate that honeybees learn to avoid extending their proboscis toward an odor associated with quinine in 1.0 M sucrose. (C) The presence of amygdalin, on the other hand, did not substantially affect acquisition during the first 3–4 trials, indicating that bees did not readily detect the toxin in the reward and instead associated the odor with sucrose. However, after the fourth trial, they began to cease exhibiting PER in response to odor at a rate that depended on the toxin dose in the reward (logistic regression: χ_1_^2^ = 77.9, p < 0.001). Note: the 0.01 mM dose was not tested for quinine; the “control” acquisition curve (1.0 M sucrose) is the same in both (A) and (C) (n = 95). n_quin_: 0.1 mM = 48, 1 mM = 27, 10 mM = 37, 100 mM = 61. n_amyg_: 0.01 mM = 80, 0.1 mM = 58, 1 mM = 57, 10 mM = 69, 100 mM = 51. (B and D) Olfactory memory consolidation was a decreasing function of toxin dose for both quinine (B) and amygdalin (D) within 10 min (colored bars) and again at 18–24 hr after conditioning (gray bars) (logistic regression: quinine: χ_4_^2^ = 167, p < 0.001; amygdalin: χ_5_^2^ = 310, p < 0.001). At the 24 hr test, the response to the odor dropped for subjects conditioned with 1.0 M sucrose (control) (χ_1_^2^ = 7.87, p = 0.005) but did not change for solutions containing quinine or amygdalin (logistic regression: quinine: χ_4_^2^ = 7.59, p = 0.108; amygdalin: χ_5_^2^ = 7.34, p = 0.194). n_quinine_: 0.1 mM = 48, 1 mM = 27, 10 mM = 37, 100 mM = 61. n_amygdalin_: 0.01 mM = 80, 0.1 mM = 58, 1 mM = 57, 10 mM = 69, 100 mM = 51. (E) Honeybees quickly learned to recognize an odor paired with sucrose and to avoid another odor paired with 1.0 M sucrose containing 10 mM quinine during a differential learning task. (F) Bees conditioned with a 1.0 M sucrose solution containing 100 mM amygdalin, however, did not readily make this distinction and stopped responding to both odors. n_quin_ = 38, n_amyg_ = 71. Error bars represent ±SEM.

Conditioned olfactory aversions to food can potentially arise by two pathways: either by association of an odor with a reflexive taste aversion toward a toxin or by association of an odor with the malaise caused by toxin ingestion. Using an olfactory conditioning paradigm for PER, honeybees were conditioned to associate an odor with either a sucrose-quinine solution or a sucrose-amygdalin solution by first touching the antenna with 1.0 M sucrose and then presenting the sucrose-toxin solution to the proboscis. When conditioned with the sucrose-quinine solution, the probability that a honeybee exhibited PER toward the odor on the third trial was 70%–90% less than its expected level for sucrose alone (Figure 2A). The subjects conditioned with the 10–100 mM quinine solutions often refused to drink; PER to the conditioned odor never rose above 40% for any of the quinine solutions, and the reduced performance persisted for 24 hr (Figure 2B). The pattern of conditioned PER when honeybees were reinforced with a sucrose-amygdalin solution was markedly different (Figure 2C). In this case, the bees learned to perform PER toward the odor during the first four trials of conditioning as if no toxin was present and rarely refused to eat the reinforcing solution. At the fifth trial (20–25 min), they began to cease responding to the odor at a rate that reflected the dose of amygdalin (logistic regression: χ_5_^2^ = 191, p < 0.001). This decline in PER arose as a result of ingesting the toxin (Figure S2A); failing to respond to the odor was also accompanied by a refusal to drink the sucrose-amygdalin solution in later trials (Figure S2B). The conditioned suppression of PER persisted for 24 hr (Figure 2D) and was specific to the conditioned odor (Figure S2C). Further evidence for the existence of two pathways comes from a comparison of the responses of honeybees during differential conditioning (Figures 2E and 2F). Honeybees differentially conditioned with sucrose and with a sucrose solution containing quinine rapidly differentiated between the two odors (logistic regression: χ_1_^2^ = 46.7, p < 0.001); those conditioned with amygdalin did not (logistic regression: χ_1_^2^ = 0.21, p = 0.646), and instead learned to avoid both odors. These data demonstrate that two pathways exist in the honeybee's brain for the learned suppression of PER: a neural circuit for associating odors with substances that evoke reflexive PER suppression and a neural mechanism that suppresses conditioned PER after toxin ingestion.

### Dopamine and Serotonin Mediate Two Pathways for Conditioned Food Aversions

Dopamine (DA) is essential for the acquisition of learned avoidances of odors signaling electric shock in insects [5, 12] and, therefore, might be expected to play an important role in conditioned food aversions in the honeybee. In nematodes, serotonin (5HT) mediates conditioned food aversions [2], but its role in olfactory learning in insects has not been established. To investigate the role of DA and 5HT as modulators of circuits involved in these two forms of learning, we used the differential conditioning assay with a sucrose-quinine solution to study the preingestive mechanism or the simple conditioning assay with an amygdalin-sucrose solution to study the postingestive mechanism.

**Figure 3:**
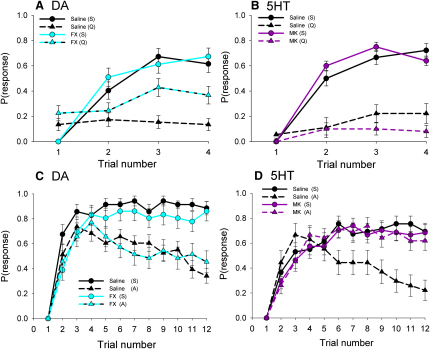
Dopamine Is Involved in Learning to Associate an Odor with a Toxin before Ingestion, but Serotonin Is Involved in Learning to Avoid a Toxin after Ingestion (A) When honeybees were injected with 0.1 mM of the DA receptor antagonist, flupenthixol (FX), they had greater difficulty learning to avoid the odor paired with a solution containing 1.0 M sucrose with 10 mM quinine during a differential learning task (n_saline_ = 52, n_FX_ = 54). The sucrose-reinforced acquisition curves for the saline and FX groups were not significantly different (logistic regression: χ_1_^2^ = 1.22, p = 0.269). (B) If injected with a 0.1 mM dose of the 5HT receptor antagonist cocktail composed of methiothepin and ketanserin (MK), honeybees performed differential learning more rapidly than the control, as indicated by the greater divergence in the purple curves on trials 3 and 4. n_saline_ = 72, n_MK_ = 40. (C) Injection with a 0.1 mM dose of the DA receptor antagonist, FX, did not impair olfactory learning toward either 1.0 M sucrose or a 1.0 M sucrose solution containing amygdalin in a simple learning task. n_saline,suc_ = 35, n_saline,amy_ = 37, n_FX,suc_ = 36, n_FX,amy_ = 34. (D) On the other hand, injection of a 1 mM dose of the cocktail of 5HT receptor antagonists (MK) abolished the ability of honeybees to learn to avoid an odor associated with an amygdalin-sucrose reinforcer. The acquisition curve produced by conditioning with the toxin after injection with the antagonist cocktail was significantly different from that for honeybees subjected to the same conditioning but injected with saline (logistic regression: χ_1_^2^ = 12.1, p = 0.001). n_saline,amy_ = 43, n_MKamy_ = 43, n_saline,amy_ = 27, n_MK,amy_ = 40. Note: the reinforcer was 10 mM amgydalin in (C) and 100 mM amgydalin in (D); both were presented in 1.0 M sucrose. See also Figure S3D. Error bars represent ±SEM.

When DA receptors were blocked with the antagonist, flupenthixol (FX), prior to differential conditioning, honeybees had greater difficulty learning to avoid the quinine-reinforced odor during acquisition (Figure 3A; logistic regression: χ_1_^2^ = 19.0, p < 0.001). We obtained similar results using a second DA receptor antagonist, butaclamol, confirming that DA is involved in this form of learning (logistic regression: χ_1_^2^ = 4.12, p = 0.042). When a cocktail of the 5HT receptor antagonists, methiothepin and ketanserin (which should block all three known invertebrate 5HT receptor classes) [13, 14], was injected prior to conditioning, the ability of bees to differentiate the outcomes associated with the two odors was slightly enhanced (Figure 3B; logistic regression: χ_1_^2^ = 9.24, p = 0.002). Specifically, the responses to the quinine-reinforced odor were on average lower for honeybees injected with the antagonists than for those injected with buffer alone (logistic regression: χ_1_^2^ = 15.7, p < 0.001), whereas the response to the sucrose-reinforced odor was unaffected (logistic regression: χ_1_^2^ = 2.57, p = 0.109).

In contrast, FX did not affect the ability of honeybees to learn to avoid an odor associated with the amygdalin solution (Figure 3C; logistic regression: χ_2_^2^ = 0.96, p = 0.327), nor did the antagonist affect a honeybee's response to the sucrose-only control (logistic regression: χ_2_^2^ = 0.31, p = 0.576). This was confirmed in separate experiments with three concentrations of the antagonist (1 to 0.01 mM; Figure S3C). On the other hand, the “inverted U-shaped” acquisition curve resulting from conditioning with a solution containing amygdalin was dramatically altered by injection with the 1 mM cocktail of 5HT receptor antagonists (Figure 3D). Injected honeybees responded to the odor as if no amygdalin was present in the sucrose solution; the acquisition curve did not differ from either of the sucrose controls (logistic regression: χ_2_^2^ = 0.52, p = 0.772), indicating that when 5HT receptors were blocked, sucrose learning proceeded as normal, but the ability to learn to associate the odor with the change in internal state arising from toxin ingestion was abolished. Injection with 0.1 or 0.01 mM concentrations of the cocktail also affected the shape of the acquisition curve (Figure S3D), but to a lesser degree over later trials. We also replicated these experiments with the octopamine (OA) antagonist, mianserin, as a control (Figures S3A and S3B); as expected, blockade of octopamine did not compromise learned avoidances of either quinine or amygdalin. Our data clearly show that although DA plays a primary role in the preingestive pathway for learned avoidances of odors, 5HT is the neuromodulator of circuits involved in the integration of postingestive signaling of malaise within the circuits governing olfactory learning in the honeybee's brain.

Our data are consistent with the possibility that a dopaminergic circuit feeds back onto the appetitive pathway for PER to establish a learned olfactory avoidance or suppression of PER when a toxin can be detected in a sucrose solution. In the worker honeybee, dopaminergic cells [15, 16] and DA receptors [17, 18] are present throughout the brain, including the mushroom bodies (MB), antennal lobe (AL), and the subesophageal ganglion (SOG). In fruit flies, DA influences learning via a subset of dopaminergic neurons that innervate the MB lobes [12] and that express two D1-like receptors [19, 20]. When DA signaling to these neurons is disrupted, appetitive olfactory learning [19, 20] and the expression of olfactory memories [21] are also reduced, indicating that DA is involved in both aversive and appetitive memory circuits in the insect MB. Furthermore, insects such as crickets fail to learn to avoid visual and olfactory cues paired with salt solutions applied to their mouthparts when DA receptors are blocked [6, 7], and larval fruit flies fail to recall learned olfactory aversions to odors paired with quinine or salt when the DA neurons in the MB are inactivated [4, 20], suggesting that DA mediates learned aversions to bad-tasting stimuli. Our data also support this hypothesis.

The primary brain regions involved in appetitive learning in the bee (the MB, SOG, and AL) are also innervated by serotonergic neurons and express 5HT receptors [22–24]. In fact, many of the 75 serotonergic neurons in the bee brain are “wide-field” neurons that innervate several brain regions [25, 26], suggesting that they have a modulatory function. Our experiments do not distinguish at which point in this pathway 5HT suppresses the expression of PER. The fact that a specific, long-term olfactory memory was formed for odors associated with ingested toxins implies that the MB is involved in this pathway [27]. A prior study found that 5HT injected into the α-lobe of the honeybee MB after conditioning significantly reduced 1 hr olfactory memory recall [28]. Alternatively, inhibition of the PER could be manifest in the neural circuitry of the SOG or the dorsal lobe, because the motor neurons that control mouthpart movements, including proboscis extension, synapse in these locations [29, 30].

Conditioned olfactory aversions to toxins based on a preingestive mechanism require the brain to form an association between two types of sensory information: odor and taste. In contrast, food aversions arising after ingestion rely on the integration of sensory information (e.g., odor) with a physiological change in state. Honeybees can learn to avoid odors using both pathways; bees could also potentially learn to associate a gustatory cue with the postingestive consequences of eating a toxin associated with it (Figure S2A) or form second-order learned associations between odor, taste, and malaise, but we did not investigate this.

At present, we do not know how sensory information is integrated with the physiological change in state arising after toxin ingestion during conditioned food aversion learning. A plausible hypothesis is that a hormone induced by physiological stress is released by the gut that targets the brain. When amygdalin is ingested by an insect, the midgut is the first area to come into contact with the toxic byproducts of its metabolism (cyanide and aglycones) [31, 32]. The endocrine cells of the midgut produce many neuropeptides that regulate feeding [33], including neuropeptide F (NPF), which is known to alter sensitivity to toxins in food in fruit flies [9]. Neurons that express NPF receptors in *Drosophila* gate the expression of appetitive olfactory memories through a subset of dopaminergic neurons that innervate the MB [21] and might also influence the circuits involved in appetitive learning. Thus, a signal released from the gut could conceivably target serotonergic neurons in the brain, causing the local release of 5HT and inhibiting PER when a toxin is detected. Alternatively, 5HT itself could be a distress signal produced by the gut. In vertebrates, at least 90% of corporeal 5HT is synthesized in the enterochromaffin cells in the gut and, when released into the blood, activates 5HT_3_ receptors in the vagal nerve to induce vomiting [34]. In insects, serotonergic cells line the gut and mesothoracic ganglia [35]. In blood-feeding insects, these cells release hormonal 5HT into the haemolymph after feeding to target 5HT receptors in other tissues such as the Malpighian tubules and salivary glands [36, 37]. If the serotonergic cells in the gut responded to toxin ingestion by releasing 5HT, they could conceivably target 5HT receptors in the brain during food aversion learning and thus provide a postingestive link between gut and brain.

Honeybees possess few gustatory receptor genes [38], and yet, like other insects, our data show that they have maintained the ability to preingestively detect alkaloids. Fewer gustatory receptors, however, may translate into reduced toxin detection for substances like amygdalin. The postingestive mechanism mediated by 5HT is likely to represent an ancestral trait maintained in animal lineages [2] and could allow honeybees to compensate for the inability to taste toxins. Although compounds such as amygdalin are occasionally found in nectar and pollen, their ecological role is not well understood [39]. One possibility is that toxins in nectar are repellent to nectar thieves such as ants but are not detected by bees. The presence of toxins in nectar could be a selected trait in this case, if the delayed action of postingestive aversive learning by bees afforded plants the short-term opportunity for pollination while avoiding the negative fitness consequences associated with a rapidly learned rejection of flowers with nectar containing an unpalatable toxin.

## Experimental Procedures

### Subjects

Worker honeybees (*Apis mellifera carnica*) were collected and restrained as described in Bitterman et al. [3] from both indoor and outdoor colonies maintained at Newcastle University or from an outdoor colony maintained at Arizona State University. Subjects that were used in the taste assay were fed 5 μl of 1 M sucrose within 30 min after restraint and then tested at least 1 hr later. Subjects were fed to satiety with 1.0 M sucrose and left for ∼24 hr prior to experimentation. The odors, 1-hexanol and 2-octanone, were used as conditioned stimuli (99.8% purity, Sigma-Aldrich). These volatile compounds have been used in previous investigations of honeybee olfactory learning [40]. Three microliter aliquots of pure odor solution were placed on a small strip of filter paper inserted into a 70 × 4 mm glass tube with plastic fittings attached at each end by silicon tubing to form an enclosed headspace. The odor tube was attached to a valve via silicon tubing that, when it was activated by a programmable logic controller (Automation Direct), shunted air through the headspace of the glass tube at 40 ml/s for 4 s.

### Taste and Mortality Assays

We developed an assay for assessing the sensitivity of the proboscis gustatory neurons to sucrose solutions containing amygdalin or quinine hydrochloride dihydrate (Sigma-Aldrich). The antennae of each bee were first briefly touched with 1.0 M sucrose to elicit proboscis extension [3]. When the proboscis was extended, a 0.6 μl droplet of 1 M sucrose was applied to the end of the mouthparts, and we recorded whether or not the subject consumed the solution. This was then repeated with a series of sucrose-toxin solutions. Each subject was fed only one dose. Prior to testing, bees were either starved for 24 hr or fed to satiety with 1.0 M sucrose. To assess the toxicity of quinine and amygdalin to bees, we fed individual bees 10 μl of sucrose-toxin solution (0.01–100 mM toxin in 1.0 M sucrose), and we counted the number of subjects that had died after 24 hr (n = 40 for each concentration of each toxin; Figure S1D). Note: several of the subjects for the 100 mM dose of quinine would not eat more than 5 μl of the solution, even when forced to do so; over 50% of these subjects died in spite of consuming half the dose.

### Electrophysiological Recordings of Gustatory Receptor Neurons in the Galeal Sensilla

Electrophysiological recordings were made from neurons located in the first ten sensilla chaetica located at the tip of the galea on the honeybee's proboscis. All stimulating solutions contained 1 mM KCl as a conductive electrolyte. A glass electrode with a tip ∼20 μm in diameter was used for both stimulating and recording [41]. Recording commenced when the open end of the glass electrode was placed over the tip of the sensillum. It was connected to a TastePROBE amplifier (Syntech) and was further amplified and filtered (CyberAmp 320, Axon Instruments; gain: 1000; eighth-order Bessel pass-band filter: 1–2800 Hz). Each stimulus trial was digitized (sampling rate 10 kHz, 16 bits; DT9803 Data Translation), stored on a computer, and then analyzed with dbWave software. Spikes were detected from a visually adjusted threshold set across the digitally filtered signal using a running median computed over 60 points. In order to sort the spikes, we used template sorting as a first approach, which was completed by a visual examination to check whether the time series extracted were consistent (see Figure S1). When multiple recordings were made for a sensillum using the same solution, the rate of spiking was averaged over all the recordings made for each GRN class.

### Electron Microscopy

Scanning electron microscopy was performed using a Cambridge Stereoscan 240 on samples that had been fixed with gluteraldehyde, washed in phosphate buffer and then in ethanol, critical point dried, and gold coated.

### Associative Conditioning

Individual, restrained worker honeybees were trained using conditioning techniques described in Bitterman et al. [3] to extend the proboscis to expect food when presented with an odor associated with a food reward. Two different conditioning paradigms with an intertrial interval of 5 min were used: (1) simple conditioning, in which one odor stimulus (conditioned stimulus, CS) was paired with an unconditioned stimulus (US; e.g., sucrose or sucrose plus toxin), on every trial for 12 trials, and (2) differential conditioning, in which two odors were conditioned on pseudorandomly alternating trials for a total of eight trials, such that each odor was associated with either sucrose alone or a sucrose-toxin solution. In the first trial of conditioning, the antennae were stimulated with a droplet of 1.0 M sucrose, and a 0.4 μl droplet of the sucrose-toxin solution was delivered to the proboscis. If a subject extended its proboscis toward the CS prior to the presentation of the US during subsequent trials, then the US was delivered directly to the proboscis; otherwise, the antennae were stimulated with 1.0 M sucrose, and the US was applied to the proboscis. Approximately 10 min and 24 hr after conditioning, each subject was presented with an unreinforced recall test with the conditioned odor and a novel odor to test for the formation of an olfactory memory.

### Pharmacological Treatments

We used DA, 5HT, and OA receptor antagonists shown in previous studies to be active against invertebrate receptors [13, 42, 43]. The DA receptor antagonists were cis-(Z)-flupenthixol dihydrochloride (Sigma Aldrich) and (+)-butaclamol hydrochloride (Sigma-RBI). The 5HT receptor antagonists were methiothepin mesylate and ketanserin tartrate (Sigma-Aldrich) used together in a cocktail of equal concentrations. The OA receptor antagonist was mianserin hydrochloride (Sigma-Aldrich). Each drug was diluted in injection saline (5 mM KCl, 10 mM NaH_2_PO_4_, pH 7.8). One microliter of drug or saline alone was injected into the brain through the median ocellus using a Hamilton syringe. Conditioning began 15–20 min after injection. Subjects were removed from the data analysis if they did not respond on any of the conditioning trials.

### Data Analysis

In the electrophysiological experiments, canonical discriminant analysis was used to compare the ratios of the GRN responses to stimuli. In the behavioral experiments, the measured response variable was whether a honeybee extended its proboscis in response to stimulation (a binary variable, yes or no). Repeated-measures binary logistic regression modeling was used to analyze response probabilities during the taste assays and during both the conditioning and test periods (SAS software, PROC GENMOD). One-tailed least-squares multiple comparison tests were conducted to make specific pairwise comparisons among test odors.

## Supplemental Information

Document S1. Three Figures and One Table
