# Supplementary material for: Parallel Reinforcement Pathways for Conditioned Food Aversions in the Honeybee
Source: Curr Biol. 2010 Dec 21;20(24):2234–40. doi: 10.1016/j.cub.2010.11.040 (PMC3011020; doi:10.1016/j.cub.2010.11.040)
Supplement: Document S1. Three Figures and One Table [file mmc1.pdf]

Current Biology, Volume *20*

Supplemental Information

Parallel Reinforcement Pathways for Conditioned Food Aversions in the Honeybee

Geraldine A. Wright, Julie A. Mustard, Nicola K. Simcock, Alexandra A.R. Ross-Taylor, Lewis D. McNicholas, Alexandra Popescu, and Frédéric Marion-Poll

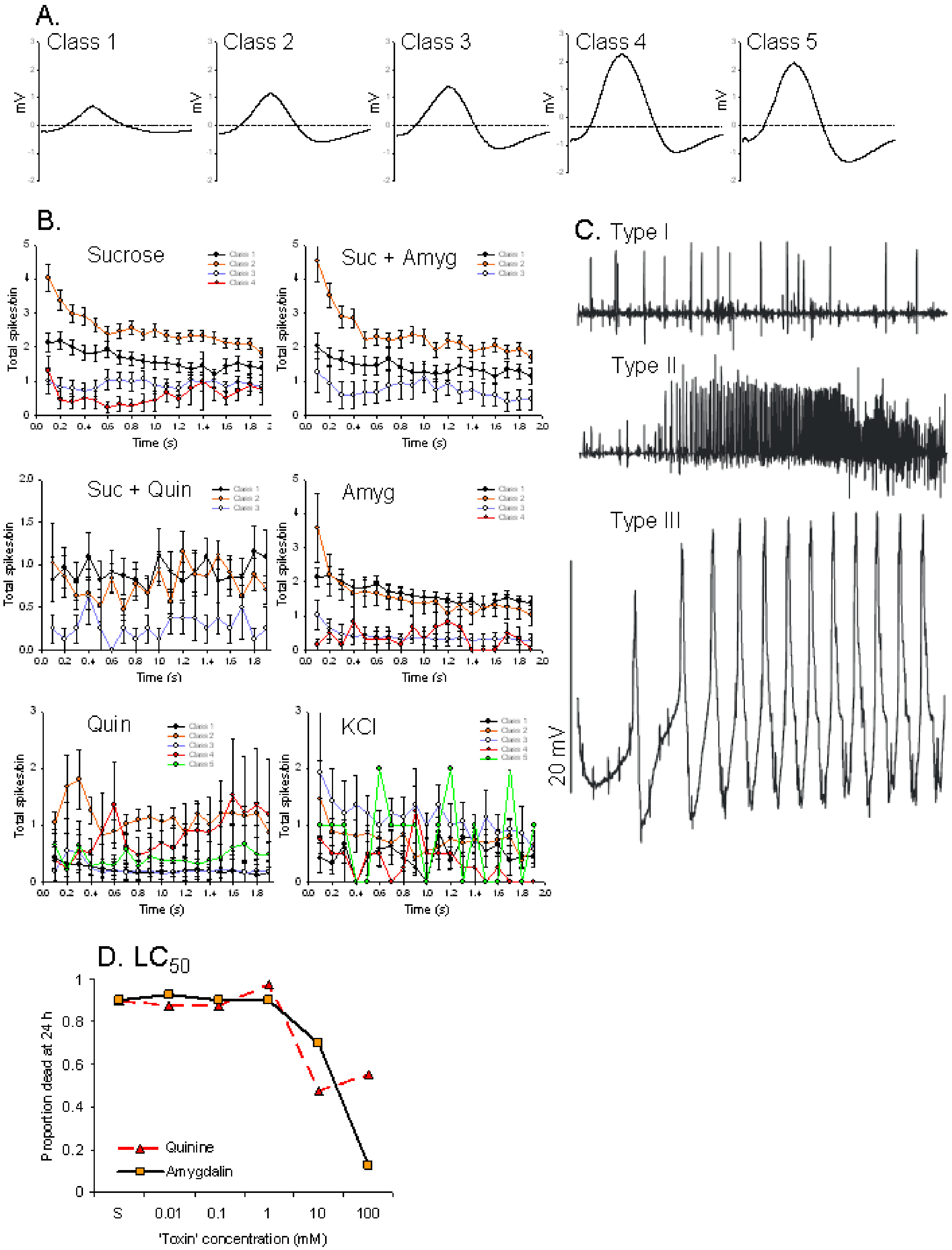
A.
Class 1
Class 2
Class 3
Class 4
Class 5
mV
B.
Sucrose
Suc + Amyg
Suc + Quin
Amyg
Quin
KCl
Total spikes/bin
Time (s)
C. Type I
Type II
Type III
20 mV
D. LC50
Proportion dead at 24 h
Quinine
Amygdalin
'Toxin' concentration (mM)
S
0.01
0.1
1
10
100

Figure S1. Responses of the Sensilla to Stimulation with Solutions Containing Quinine and Amygdalin (Related to Figure 1)

(A) Five neuron 'classes' were identified from the spike sorting for the traces generated from stimulation with the 6 substances. Each figure represents the average waveform computed from the identified spikes for each class. Spike sorting was first accomplished by first setting a threshold across a digitally filtered signal computed from the running average of 60 points around the spike (see Fiore, L., Corsini, G., and Geppetti, L. 1996. *J. Neurosci. Methods 70*, 177-184.) These spikes were then sorted using a template sorting approach described by Smith, J.J.B., Mitchell, B.K., Rolseth, B.M., Whitehead, A.T, and Albert, P.J. 1990. *Chem. Sens.* 13: 253-270.

(B) Peristimulus time histograms for the responses of each class of neuron illustrate that sucrose solutions and solutions containing amygdalin produce mainly phasic responses in the GRNs while quinine produces tonic responses. Note: y-axis for each graph is not the same.

(C) Electrophysiological recordings of the responses of GRNs housed in sensilla located on the honeybee's galea during stimulation with 10 mM quinine over a 2 s interval. Type I responses to quinine: a subset of sensilla (14 out of 45) responded with a tonic firing rate. Type II: a subset of sensilla (19 out of 45) responded with a classic 'deterrent' cell response in which the rate of firing starts low and ends in high rate of bursting. Type III: a subset of the sensilla (12 out of 45) responded with oscillations in voltage which exceeded 25 mV.

(D) The LC_{50} (lethal concentration for 50% of the population) calculated for each concentration of quinine and amydalin (in 1.0 M sucrose) after 24 h based on an N = 40 for each point. Note: for the 100 mM quinine, no bees consumed over 5 μl of the 10 μl dose. Error bars represent $\pm$ SEM.

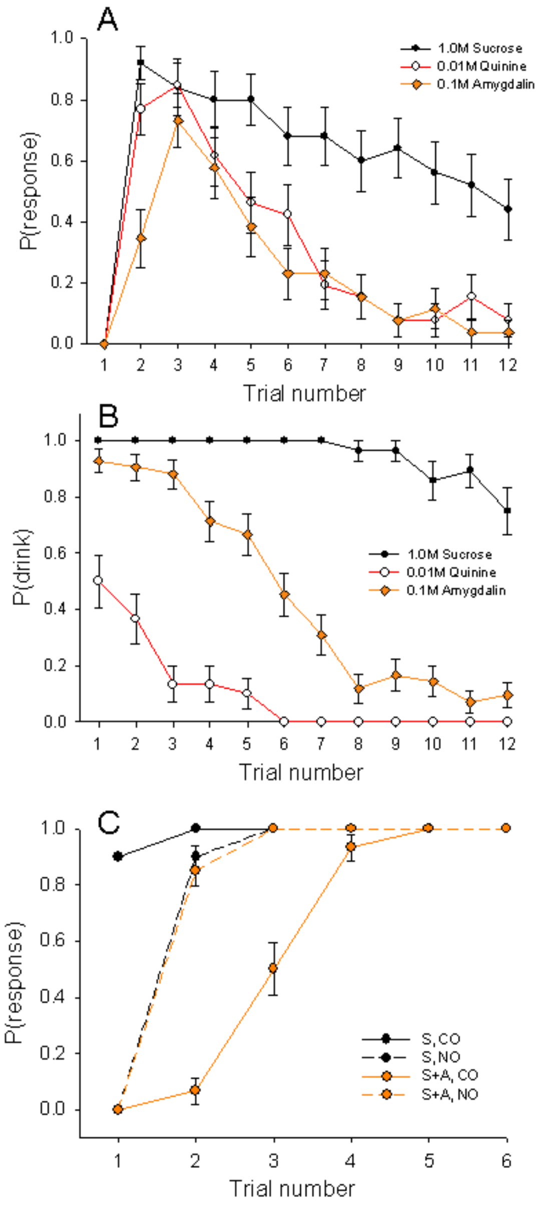

Figure S2. Learning to Avoid an Odor Paired with a Solution Containing Toxin Arises from a Post-Ingestive Mechanism that Produces a Long-Term Memory Specific to the Conditioned Odor (Related to Figure 2)

(A) An experiment was designed to dissociate the timing of the odor-reward contingency from the ingestion of toxin to confirm the involvement of a post-ingestive mechanism during olfactory aversion learning. Honeybees were fed 1μl of one of three solutions (1.0 M sucrose, 1.0 M sucrose with 10 mM quinine, or 1.0 M sucrose with 100 mM amygdalin) five min after each conditioning trial of odor paired with 1.0 M sucrose. Honeybees learned to avoid the odor paired with 1.0 M sucrose as if the reward contained the toxin, indicating either that post-ingestive feedback arising from toxin consumption occurring between trials was influencing olfactory learning during acquisition. (Alternatively, this data could also show that honeybees can form a second-order learned association

between an odor, a gustatory cue (e.g. the taste of sucrose) and the post-ingestive consequences of eating a toxin.) Bees fed solutions containing either quinine or amygdalin exhibited this effect. Being fed 1.0M sucrose between trials also gradually reduced their rate of response to the conditioned odor, but at a much slower rate than those fed the toxins (logistic regression χ_{22}^2 = 51.4, p < 0.001).

(B) The willingness of honeybees to consume the amygdalin dose fed between the conditioning trials in (A) decreased as a function of trial number. The honeybees fed the solution containing quinine often refused to consume the entire dose and were force-fed each time. A small proportion of those fed 1.0 M sucrose also began to refuse to drink sucrose towards the end of the conditioning period. For both A and B: N_{suc} = 28, N_{quin} = 30, N_{amyg} = 42.

(C) The post-ingestive mechanism for learned food aversions produced a specific, long-term memory for the conditioned odor (CO). To confirm this, an additional reversal learning test was performed at 24 h after conditioning that examined whether a honeybee had learned specifically to avoid the CO. Subjects that had previously been conditioned with either a 1.0M sucrose solution or a 1.0 M sucrose solution containing 100 mM amygdalin were subsequently conditioned with 1.0 M sucrose paired either with the original CO or with a novel odor (NO) approximately 24 h later. Ninety percent of subjects originally conditioned with sucrose solution responded to the CO on the first trial; those conditioned with the NO learned the task within 3 trials. Both the sucrose-only and the amygdalin pre-conditioned groups learned to associate the NO with the sucrose solution at an equal rate (Poisson regression: χ_1^2 = 0.03, p = 0.871). Those originally conditioned with the sucrose-amygdalin solution, however, experienced more difficulty learning to associate the CO with sucrose than the NO group (Poisson regression: χ_3^2 = 20.2, p < 0.001). The reduction in the rate of acquisition for this group indicates that honeybees had learned specifically to associate the CO with the ingestion of toxin. $N_{suc, CO}$ = 30, $N_{suc, N}$ = 28, $N_{amy, CO}$ = 32, $N_{amy, N}$ = 30. Error bars represent $\pm$ SEM.

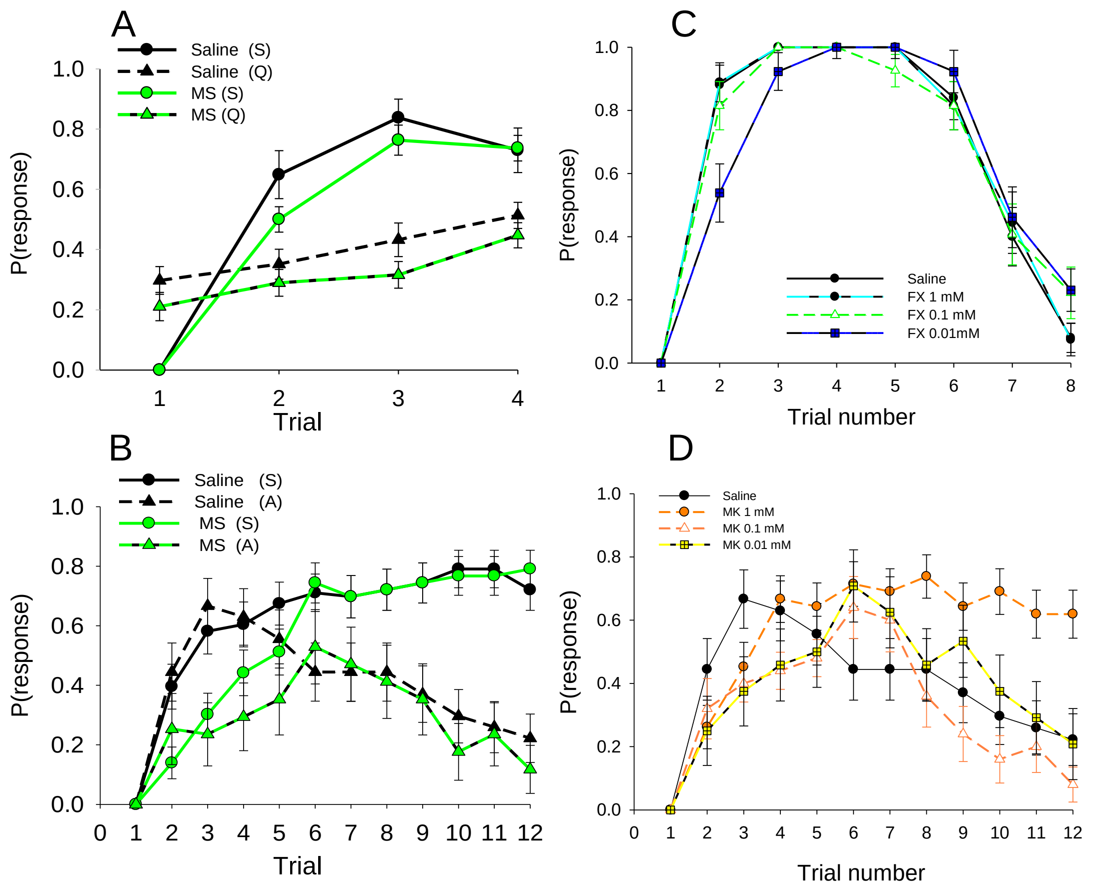

Figure S3. Confirmation of Effect of Octopamine, Dopamine and Serotonin Receptor Antagonists on Learned Olfactory Avoidances of Quinine and Amygdalin (Related to Figure 3)

A series of control experiments with the antagonist for the neurotransmitter, octopamine (OA), were performed to confirm that OA signalling was not involved in aversive olfactory learning.

(A) A 100 mM dose of the OA receptor antagonist, mianserin (MS), reduced the responses towards both the odor associated with 1.0 M sucrose and the odor associated with the 1.0 M sucrose solution containing 10 mM quinine during differential conditioning $N_{saline} = 37$, $N_{MS} = 38$.

(B) A 0.1 mM dose of the OA receptor antagonist, mianserin (MS), affected simple olfactory learning during the first 5 trials for odors paired with 1.0 M sucrose and odors paired with 1.0M sucrose containing 100 mM amygdalin, but it did not affect the levels of response during the remaining 7 trials with either reward. $N_{saline,suc} = 46$, $N_{saline,amy} = 45$, $N_{MS,suc} = 32$, $N_{MS,amy} = 18$.

(C) To confirm that the dopaminergic antagonist, flupenthixol (FX), did not affect a honeybee's ability to avoid a 1.0 M sucrose solution containing 10 mM amygdalin, three concentrations of the drug were tested. Acquisition was not significantly different for any of the treatments, including the saline control (logistic regression $\chi_3^2 = 4.30$, p = 0.231), confirming that dopamine is not involved in the post-ingestive component of aversive olfactory learning. N_{flu}: 0.01 mM = 28, 0.1 mM = 27, 1 mM = 27, $N_{saline} = 30$.

(D) Three concentrations of the 5HT antagonist cocktail (methiothepin and ketanserin, MK) were tested to confirm that 5HT was involved in the post-ingestive pathway for learning to avoid odors paired with a sucrose solution containing amygdalin. The effect of the cocktail was dose-dependent. If honeybees were injected with the 0.1 mM concentration, the acquisition curve was different from

the control: the rate of acquisition during the first four trials was reduced (χ_1^2 = 5.88, p = 0.015), but the response expected during trials 6 and 7 was significantly greater than that for the control (χ_1^2 = 5.59, p = 0.018). The last five trials were not significantly different from the control (χ_1^2 = 0.39, p = 0.531). Acquisition after injection with 0.01 mM was not significantly different to that produced after injection with 0.1 mM (logistic regression: χ_1^2 = 2.86, p = 0.091). The saline control and the 1 mM concentration were included for comparison (also shown in Figure 3). N_{MK}: 10^{-5}M = 24, 10^{-4}M = 25, 10^{-3}M = 40, N_{saline} = 27. Error bars represent ± SEM.

Table S1. A Canonical Discriminant Analysis Classified the Stimuli Applied to the Honeybee's Galeal Sensilla by the Ratio of the Rate of Firing of 5 Classes of Gustatory Receptor Neurons

	**Discriminant Function Number**				
	**1**	**2**	**3**	**4**	**5**
**% Variance**	71%*	25.5%*	2.3%	1.1%	0.1%
**S**	**0.782**	0.156	0.007	0.056	-0.020
**S + A**	**0.559**	-0.011	-0.020	-0.037	0.037
**S + Q**	-0.806	-0.551	-0.156	0.087	0.000
**A**	-0.270	-0.268	0.014	**-0.213**	-0.021
**Q**	**-1.271**	**1.189**	-0.052	-0.005	0.003
**KCl**	-0.822	-0.280	**0.432**	0.076	0.007
	**Standardized Canonical Discriminant Function Coefficients**				
**Function**	**1**	**2**	**3**	**4**	**5**
**GRN1**	0.721	0.229	-0.341	0.671	0.021
**GRN2**	0.826	0.340	0.042	-0.548	0.069
**GRN3**	0.161	0.104	0.843	0.560	-0.171
**GRN4**	-0.307	0.724	-0.240	-0.070	-0.582
**GRN5**	-0.258	0.613	-0.007	0.071	0.751

Two of 5 discriminant functions significantly separated the stimuli into subsets (* indicates that the function was classified the stimuli with $p < 0.05$). The magnitude and direction of the unstandardized coefficients of the first function reveal that the responses to S (300 mM sucrose) and S + A (300 mM sucrose with 10 mM amygdalin) are significantly different from those produced towards all the other stimuli and most different from those produced by quinine alone. The rank of similarity described by Function 1 is as follows: S, S+A, A, S + Q, KCl, Q. This function accounts for 71% of the variance in the data. The second function, which accounts for 25% of the variance, significantly separates Q (0.01M quinine) from all the other stimuli. The standardized coefficients reflect how activity in specific neurons contributes towards the classification of the responses by stimulating solution.
